# Editorial: Motion tracking and deformation analysis in biomechanics

**DOI:** 10.3389/fbioe.2025.1691342

**Published:** 2025-09-08

**Authors:** Jiaqiu Wang, Chi Wu, Qiuxiang Huang, Ran He, Zhiyong Li, Liguo Zhao

**Affiliations:** ^1^ School of Computer Science and Digital Technologies, London South Bank University, London, United Kingdom; ^2^ School of Mechanical, Medical and Process Engineering, Queensland University of Technology, Brisbane, QLD, Australia; ^3^ Centre for Biomedical Technologies, Queensland University of Technology, Brisbane, QLD, Australia; ^4^ School of Engineering, University of Newcastle, Callaghan, NSW, Australia; ^5^ School of Engineering, University of Leicester, Leicester, United Kingdom; ^6^ Faculty of Sports Science, Ningbo University, Ningbo, Zhejiang, China; ^7^ School of Energy and Power Engineering, Nanjing University of Aeronautics and Astronautics, Nanjing, Jiangsu, China

**Keywords:** biomechanics, kinematics, deformation, motion tracking, finite element analysis, computational model, ultrasound, optical coherence tomography

## Introduction

Biomechanical analysis plays a critical role in the clinical diagnosis and treatment of a wide range of diseases. At the whole-body level, it is essential for assessing movement, exercise performance, and sports biomechanics. At the tissue level, deformation and strain characteristics are closely linked to pathological changes in conditions such as cancer, cardiovascular disease, musculoskeletal disorders and etc.

This Research Topic aims to explore the forefront of innovative imaging, sensing, and computational modelling techniques in biomechanical analysis. It particularly highlights cutting-edge research that utilises non-invasive, contactless technologies and advanced algorithms to uncover biomechanical characteristics. Special emphasis is placed on the integration, clinical application, and validation of these methods using real-world patient data.

This Research Topic collects 16 high-quality publications, including one review article and 15 original research papers. Based on the focus areas, the contributions can be broadly categorised into the following groups: (1) biomechanics in sports and performance analysis; (2) kinematics and kinetics in rehabilitation evaluation; (3) musculoskeletal dynamics; (4) finite element analysis (FEA) in bone and joint; (5) biomechanical measurements in vascular system.

## Biomechanics in sports and performance analysis

Biomechanics, along with relevant imaging, sensing, and modelling techniques, provides the foundation for assessing human physical performance. These approaches help individuals improve their exercise outcomes and show great potential in enhancing athletes’ professional skills in competitive sports.

Huang et al. presented an intriguing study sparked by the fusion of modern biomechanical analytical technologies with the traditional Chinese martial art of Tai Chi. Using measurement platforms for three-dimensional (3D) motion capture, force analysis, and electromyography, the study quantitatively investigated the correlations among kinematic, kinetic, and surface electromyography (sEMG) characteristics, as well as the stability index, during the balance phase of 143D, one of the most challenging movements in competitive Tai Chi. This work advances the understanding of movement characteristics and balance mechanisms from the biomechanical perspective, providing theoretical support to enhance training effectiveness and athletic performance.

Wang et al. investigated dynamic balance during step descent in 20 young women using F-Scan plantar-pressure insoles across four heights (5, 15, 25, 35 cm). They quantified landing strategy and center-of-pressure (COP) metrics and analyzed step height. With increasing height, COP velocities and ranges rose significantly (p < 0.001), indicating greater balance demand; preferred landing shifted from hindfoot toward forefoot beginning at 5 cm. Dominant vs. non-dominant leading foot showed similar kinetics, except a smaller 95% COP area for the dominant foot (better stability; p = 0.013). The study informs a practical stair design for fall-prevention guidance.

The study by Huang et al. investigated the effects of hammer rotation on the performance in hammer throwing of female athlete. By comparing each athlete’s long and short competition trials and employing three-dimensional motion analysis, they found that longer throws featured greater release velocity, higher velocity gain during double support, shorter single-support phases, lower horizontal azimuth angles, and longer rotational radii at key instants. These findings offer insights for enhancing biomechanical efficiency and training strategies in the hammer throwing competition.

## Kinematics and kinetics in rehabilitation evaluation

In rehabilitation, biomechanical forces are key parameters for ensuring optimal outcomes, particularly for exercise-related conditions. Advanced biomechanical analytical approaches greatly enhance the effectiveness of rehabilitation through deeper insights and more precise interventions.

Orthopaedic insoles are commonly used to address flatfoot conditions, but their biomechanical effectiveness across different movements remains debated. Chen et al. published a systemic review exploring the impact of orthopaedic insoles on the kinematics and kinetics of lower limb motion, with a focus on evaluating their effectiveness and identifying future research directions in flatfoot biomechanics. The review comprehensively analyses 19 studies filtered from 671 screened cohorts, and confirms the effectiveness of orthopaedic insoles in improving movement patterns in patients with flatfoot. It also highlights several current limitations and key directions for future research, including the need for further investigation into biomechanical effectiveness, the potential of personalised orthopaedic insoles, and the importance of conducting studies across diverse populations and movement types, as well as long-term interventions.

Ruan et al. used dynamic biplane radiography to assess ankle joint kinematics and ligament deformation in 12 patients with chronic ankle instability (CAI) during walking. Compared with the uninjured side, CAI ankles showed reduced tibiotalar dorsiflexion, greater talar displacement, and increased subtalar inversion. During stance, the anterior talofibular ligament (ATFL) elongated with plantarflexion and talar anterior translation, while the calcaneofibular ligament (CFL) shortened. The findings highlight the importance of sagittal alignment in ligament repair and underline biomechanical markers for targeted rehabilitation aimed at restoring balanced ankle stability in CAI patients.

Kong et al. presented a study involving a cohort of 49 patients who underwent anterior cruciate ligament reconstruction (ACLR), aiming at exploring dynamic changes in six degrees of freedom (6DoF) knee joint kinematics over a 1-year rehabilitation period. By combining kinematic analysis with clinical scoring systems, the study investigated functional recovery patterns and provided evidence to support the optimisation of postoperative rehabilitation strategies. The findings highlight the importance of individualised rehabilitation, emphasising joint stability and range of motion recovery during the early postoperative phase (0–6 months), and correction of rotational and flexion-extension functions during the later phase (6–12 months) to enhance knee function and prevent long-term adverse outcomes.

## Musculoskeletal dynamics

The musculoskeletal system provides humans with the power of motion. Although the underlying processes are highly complex, modern imaging and modelling techniques allow these processes to be quantitatively revealed and analysed.

Sewify et al. explored the feasibility of combining 3D ultrasound and motion capture for non-invasive tracking of shoulder bones. Through *in silico* simulations and cadaveric experiment, they compared two registration algorithms: Iterative-Closest-Point (ICP) and Coherent Point Drift (CPD), for aligning full bone models with 3D ultrasound-imaged surface patches. CPD showed higher accuracy but longer computation time, suggesting it may be more suitable for this task. The study highlights the promise of 3D ultrasound with motion capture for dynamic bone tracking.

Sahrmann et al. introduced a 3D ultrasound-based method to quantify shape deformations of the tibialis anterior (TA) muscle during dynamic contractions. They found that muscle volume remained constant across different muscle lengths, while mean cross-sectional area (CSA) increased during TA muscle shortening. Additionally, maximum CSA shifted proximally with shortening. Significant differences were found between active and passive movements, especially at higher velocities. Their approach enables non-invasive, dynamic tracking of muscle morphology and provided new insights for future empirical studies and musculoskeletal modelling.

From the perspective of energy and power, Stróżyk and Bałchanowski analysed unilateral food chewing, a process involving complex biomechanical interactions within the masticatory system. Using experimentally derived food resistance patterns and a 3D kinematic-dynamic model of the masticatory system, they simulated unilateral chewing to quantify energy and peak power generated by the masseter, medial pterygoid, and temporalis muscles. This study provides biomechanical insights into mastication: food height and texture influence muscle energy demands, with greater energy from the masseter and medial pterygoid on the working side and higher temporalis activity on the non-working side. Food position more strongly affects peak muscle power for foods with high texture heterogeneity.

## Finite element analysis in bone and joint

Finite element analysis (FEA) is a powerful numerical method originally developed in engineering to solve mechanical problems. Applied to biomechanics, it has proven highly effective in analysing intricate biomechanical forces and deformations, particularly in scenarios such as disease condition assessment, treatment planning, and bespoke implant design.

Luo et al. used FEA to investigate the effects of different manipulations of lower limb hyperextension (MLLHs) on the sacroiliac joint (SIJ) and surrounding ligaments. A 3D pelvis model was constructed, and four types of MLLHs were simulated. The resulting stresses on the pelvis and SIJ, as well as SIJ displacements and ligament strains, were analysed to quantitatively characterise the biomechanical effects of MLLHs and to provide a reference for clinical practice.

Chen et al. conducted a FEA to simulate two different fixations, the long-segment spinal inner fixation (LSIF) and the tandem spinal external fixation (TSEF) in treating multilevel non-contiguous spinal fracture (MNSF), evaluating their biomechanical performance. They found that TSEF demonstrated outperformed biomechanical characteristics compared to LSIF, including better preservation of range of motion and more balanced disc stress distribution. TSEF also reduced stress concentration on connecting rods and minimized stress shielding of fractured vertebrae, suggesting it may be a more effective alternative for managing MNSF.

Roland et al. performed FEA on a fractured human tibial shaft to identify optimal axial stroke values for “smart” fracture plates that were capable of cyclic shortening/lengthening. In the study, the FE models were reconstructed from CT data and incorporated virtual implants, simulating various fracture gap sizes (1–3 mm), angles (5°–60°), and healing stages. Mechanical parameters, including strain measures and the “perfect healing window”, were calculated and examined using linear regression. The analyses identified optimal strokes of 0.10–0.25 mm in early healing, 0.10 mm in mid-healing, and 0.35–0.45 mm in late healing, with gap size showing the greatest influence. These findings offer quantitative design guidance for active implants to enhance fracture healing.

In another FEA-based study, Luo et al. evaluated the biomechanical effects of varying microfracture drilling parameters, including diameter, depth, and spacing, on bone stability at cartilage defect sites. A femoral medial condyle defect model with different drilling configurations was subjected to FEA under simulated standing-load conditions. The results indicated that all parameter combinations produced stresses below the bone’s yield strength, with smaller diameter drills and closer spacing yielding the lowest Von Mises stresses. These findings provide biomechanical insight to support the selection of optimised drilling parameters in microfracture surgery, helping to preserve bone stability.

## Biomechanical measurements in vascular system

As a whole-body system, vascular system is responsible to provide sources of pump blood to whole body. Its complexity is reflected in its intricate network, multi-level structure, and variations in flow, velocity, and pressure at different locations. Moreover, the interaction between pulsatile blood flow and the compliant vessel walls poses additional challenges for performing accurate biomechanical measurements in the vascular system.

Curcio et al. evaluated non-invasive biomechanical metrics, including ultrasound-based point shear wave elastography (p-SWE) and wall tracking stiffness assessment, as well as computed tomography angiography (CTA)-derived 3D modelling with patient-specific FEA, to classify vulnerable carotid plaques in 100 endarterectomy patients. Vulnerable plaques showed significant differences in both ultrasound-derived stiffness measures, such as Young’s modulus, and FEA-based stress parameters, including von Mises and maximum principal stress patterns. Both modalities effectively differentiated vulnerable from stable plaques, supporting their potential for non-invasive stroke risk assessment.

Zhang et al. developed an objective optical coherence tomography angiography (OCTA) workflow for quantifying oral microvasculature. They employed a swept-source OCT system to scan 37 volunteers across four intraoral sites and analyzed a benign ulcer. They introduced automatic depth-of-interest segmentation and four metrics, namely, vessel area density (VAD), vessel skeleton density (VSD), vessel diameter index (VDI), and a novel weighted tortuosity index (WTI). Repeat scans showed low coefficients of variation, and site-specific patterns emerged (e.g., hard palate: lower VAD/VSD; floor of mouth: larger VDI). The work establishes reproducible OCTA metrics and WTI for early diagnosis and monitoring.

Wei et al. validated a porous-medium (PM) model for improving accuracy of patient-specific computational fluid dynamics (CFD) results in analysing iliac vein compression syndrome (IVCS). They reconstructed bilateral iliac veins with collaterals, modelled laminar Newtonian blood flow, tuned PM viscous resistance, and used a discrete-phase model to reproduce Digital Subtraction Angiography (DSA) contrast transit via time-to-peak (TTP). The PM model transported over 80% of particles through collaterals and matched DSA TTP (92.4% concordance). Compared to a non-porous model, the proposed PM model cut stenotic-region velocity by 87.5%, increased the IVC–left iliac pressure gradient by 141 Pa, and shifted WSS >2.0 Pa to collateral vessels. The approach aligns CFD with clinical hemodynamics and supports IVCS assessment and treatment planning.

## Outlook

This Research Topic integrates the latest advancements in deformation and biomechanical analysis, spanning from whole-body to tissue-level studies. It highlights innovations in imaging, sensing, computational analysis, and modelling techniques, and further identifies current limitations while outlining future research directions. Collectively, These contributions underscore the significance of the field in advancing toward real clinical impact.

